# Multiomic profiling reveals timing of menopause predicts prefrontal cortex aging and cognitive function

**DOI:** 10.1111/acel.14395

**Published:** 2024-11-05

**Authors:** Fatima Gunter‐Rahman, Charleen D. Adams, Ravikiran M. Raju, Yu Zhang, Eunjung Alice Lee, Carmen Messerlian

**Affiliations:** ^1^ Harvard‐MIT Program in Health Sciences and Technology Massachusetts Institute of Technology Cambridge Massachusetts USA; ^2^ Division of Genetics and Genomics Boston Children's Hospital Boston Massachusetts USA; ^3^ Department of Environmental Health Harvard T.H. Chan School of Public Health Boston Massachusetts USA; ^4^ Division of Newborn Medicine, Boston Children's Hospital Harvard Medical School Boston Massachusetts USA; ^5^ Picower Institute for Learning and Memory Massachusetts Institute of Technology Cambridge Massachusetts USA; ^6^ Department of Pediatrics Harvard Medical School Boston Massachusetts USA; ^7^ Broad Institute of MIT and Harvard Cambridge Massachusetts USA; ^8^ Department of Epidemiology Harvard T.H. Chan School of Public Health Boston Massachusetts USA; ^9^ Department of Obstetrics and Gynecology, Vincent Center for Reproductive Biology Massachusetts General Hospital Fertility Center Boston Massachusetts USA

**Keywords:** aging, dementia, menopause, multiomics

## Abstract

A new case of dementia is diagnosed every 3 s. Beyond age, risk prediction of dementia is challenging. There is growing evidence of underlying processes that connect aging across organ systems and may provide insight for early detection, and there is a need to identify early biomarkers at an age when action can be taken to mitigate cognitive decline. We hypothesized that timing of menopause, a marker of ovarian aging, predicts brain age decades later. We used 2086 subjects with multiple “omics” measurements from post‐mortem brain samples. Age at menopause (AAM) is positively correlated with cognitive function and negatively correlated with pre‐frontal cortex aging acceleration (calculated as estimated biological age from DNA methylation minus chronological age). Genetic correlations showed that at least part of these associations is derived from shared heritability. To dissect the mechanism linking AAM to cognitive decline, we turned to transcriptomic data which confirmed that later AAM was associated with gene expression in pre‐frontal cortex consistent with better cognition, and among those who reached menopause naturally, decreased gene expression of pathways implicated in aging. Those with surgical menopause displayed different molecular changes, including perturbed nicotinamide adenine dinucleotide (NAD+) activity, validated by metabolomics. Bile acid metabolism was perturbed in both groups, although different bile acid ratios were associated with AAM in each. Together, our data suggest that AAM is predictive of brain aging and cognition, with potential mediation by the gut, although through different mechanisms depending on the type of menopause.

## Graphical abstract

Age at menopause is positively correlated with cognitive function and negatively correlated with prefrontal cortex aging acceleration. Transcriptomic and metabolomic data from the brain show evidence of involvement of the gut, and both shared and different pathways by type of menopause (surgical vs. natural). This highlights a connection of aging across organ systems and shows that age at menopause can be used to inform brain aging decades in advance.

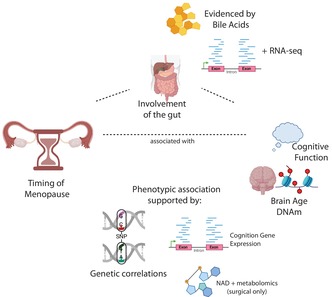

**Abbreviations**
AAM: Age at Menopause
AD: Alzheimer's disease
APOE: apolipoprotein E
BA: Broadman Area
BMI: Body Mass Index
CA: cholic acid
CDCA: chenodeoxycholic acid
CDR: Clinical Dementia Rating
COG‐NEG: the top 500 genes by p‐value that were negatively correlated with cognition
COG‐POS: the top 500 genes by p‐value that were positively correlated with cognition
DCA: deoxycholic acid
FDR: false discovery rate
fGSEA: fast Gene Set Enrichment Analysis
GAMs: genes associated with continuous AAM
GO:MF: gene ontology: molecular function
GSEA: Gene Set Enrichment Analysis
GWAS: genome‐wide association study
HRT: hormone replacement therapy
IGF: insulin‐like growth factor
LCA: lithocholic acid
LD: linkage disequilibrium
MAP: Memory and Aging Project
miRNA: microRNA
MRC IEU: Medical Research Council, Integrative Epidemiology Unit
MSBB: Mount Sinai Brain Bank
mTORC: mammalian target of rapamycin complex
NAD+: nicotinamide adenine dinucleotide
NR: nicotinamide riboside
ROS: Religious Order Study
UKBB: United Kingdom Biobank

## 1 INTRODUCTION

Life expectancy has increased dramatically over the last century; however, morbidity, including dementia, severely impairs quality of life in the elderly. There is strong evidence for reproductive and sex‐specific modifiers of aging. Among markers of reproduction, age at menopause (AAM) is of particular interest due to its ease of measurement and use as a marker of a key life‐stage transition. AAM is approximately 50% heritable (Murabito et al., 2005), and associated loci are in DNA‐damage response pathways (Ruth et al., 2021a). Earlier age at menopause (AAM) is associated with both aging‐related conditions (e.g., frailty, cardiovascular disease, and all‐cause mortality) (Davis et al., 2015; Mason et al., 2022; Verschoor & Tamim, 2019), and molecular markers of aging: increased age acceleration calculated using DNA methylation clocks (Levine et al., 2016) and decreased telomere length (Gray et al., 2014; Schuermans et al., 2023). Relatedly, infertility is associated with premature mortality (Wang et al., 2022). Animal studies also demonstrate a relationship between reproduction and longevity: older mice in which young ovaries are transplanted show a significant increase in lifespan (Mason et al., 2009). Some have suggested that menopause itself accelerates biological aging (Levine et al., 2016), but it is also possible that accelerated aging across the body impacts reproductive longevity. Some factors that potentially affect accelerated aging include obesity and smoking, as they are associated with both earlier menopause and increased risk of dementia (Davis et al., 2015; Mason et al., 2022; Whitmer et al., 2008; Zhong et al., 2015).

Dementia is one of the major concerns regarding an aging population. The brain typically displays signs of aging after age 70, which is often too late to slow down or reverse degeneration. The relationship between reproductive aging and brain aging remains unclear. A meta‐analysis of 13 studies found no association between AAM and incidence of dementia (Georgakis et al., 2016), but a recent, much larger study found that later AAM was associated with a lower risk of dementia, only among those with natural menopause (Hao et al., 2023). Another study confirmed a relationship between AAM and cognitive function scores among those with natural menopause and also found increased brain volume associated with later AAM (Costantino et al., 2023). This conflicts with a previous study that found a relationship between cognitive function and AAM only among those with surgical menopause (Bove et al., 2014). Possible explanations for these discrepancies include insufficient accounting of potential confounders, lack of statistical power, heterogeneity of conditions that all bear the label dementia, and different ages at measurement. Moreover, there is a lack of genetic and molecular characterization underlying these associations.

To better understand the relationship between AAM and cognition, we utilize longitudinal cognitive test scores in the elderly across various cognitive domains from two large, national United States cohort studies: the Religious Order Study (ROS) and Memory and Aging Project (MAP) (Bennett et al., 2018a, 2018b). This is more sensitive than a binary categorization of dementia or no dementia, and also provides details on cognition at different ages. To understand the relationship between AAM and brain aging, we rely on DNA methylation from the brain to calculate epigenetic brain age acceleration, and look at its association with AAM. We then delve into genetic, transcriptomic, and metabolomic data to dissect the mechanism underlying the phenotypic correlations observed, separately in natural menopause and surgical menopause (oophorectomy or hysterectomy before natural menopause), as mechanisms in each group may differ. The integration of multiomics data, including bile acids and other metabolomics data, allows us, for the first time, to investigate the possible mechanisms underlying the association between AAM and brain aging, in natural and surgical menopause populations separately. Despite differences in mechanism between natural and surgical menopause associated cognitive effects, our results demonstrate that AAM can be used as a biomarker for accelerated aging that extends to cognition and brain aging.

## 2 METHODS

### 2.1 Analysis of cognition scores

Participants in ROS and MAP (Bennett et al., 2018b) cohorts undergo 19 cognitive tests at baseline and each follow‐up, including the East Boston immediate and delayed recall, reading tests, and stroop tests. A full list of tests can be found in the Rush Alzheimer's Disease Center documentation (Table S1). Global cognition scores, as well as cognitive subdomain scores, are calculated as an average of normalized scores (z‐scores). We excluded participants with missing data or with AAM before 30 or AAM after 60 (Figure S1). We tested for the association between AAM and global cognition scores at ages 65, 70, 75, 80, 85, 90, and 95 using separate linear regressions (base R 4.1.0 (R Core Team, 2021) linear regression) adjusting for age at menarche, body mass index (BMI), lifetime daily alcohol intake (categorized from 1 to 9), smoking status (never, former, and current), study cohort (ROS vs. MAP), carrier of apolipoprotein E (APOE) E4 allele (yes vs. no), and education (total number of years). We tested for an association of each cognitive subdomain score at age 70 and AAM with the same potential confounders. E‐values, which measure the size of an association an unmeasured confounder would need to have with both the exposure and the outcome to fully explain the relationship (VanderWeele & Ding, 2017), were calculated based on the regression from age at menopause and cognition at age 70, 80, and 90, with all previously mentioned covariates. Due to inconsistent reporting, we held a stringent standard for the hormone replacement therapy (HRT) negative analysis. We excluded participants whose response to HRT use was ever coded as “yes” or “suspect”, in the baseline assessment or in any follow‐up year. Other sensitivity analyses were restricted to the respective group as described (e.g., ROS cohort only).

Linear mixed models were used to investigate the association between AAM and longitudinal cognition over time, using an interaction term of AAM and age in years after 65 (both continuous). Random intercepts and random slopes were based on individual participant IDs (“projid”). Natural and surgical menopause groups were analyzed separately. For the fixed effects, we included the same covariates as the cognition at ages 65–95 above, and age at enrollment.

### 2.2 DNA methylation analyses

DNA methylation profiles from Illumina HumanMethylation450 BeadChip from post‐mortem prefrontal cortex samples were previously generated and processed as a part of the ROSMAP samples as described (De Jager et al., 2014). Estimates of biological brain age were calculated using CorticalClock (Shireby et al., 2020) (code available https://github.com/gemmashireby/CorticalClock). We used a generative additive model (R package mgcv) to test for a nonlinear relationship between biological age and chronological age. We used Pearson correlation to examine the relationship between biological age and cognition score at last visit before death. Age acceleration was calculated as biological age—chronological age (age at death), and Pearson correlation was used to examine the relationship between age acceleration and AAM. To account for covariates, a generalized additive model was used to test for an association between predicted brain age and AAM, adjusted for the same covariates as cognition score analyses, plus a nonlinear term for age at death, because we found a significant non‐linear relationship as described above.

### 2.3 Genetic correlations

Genetic correlations between measures of AAM and two measures of Alzheimer's disease (AD)/dementia and two measures of accelerated biological age were obtained by integrating genetic data from genome‐wide association study (GWAS) summary statistics from eight studies. Four were downloaded from the Medical Research Council, Integrative Epidemiology Unit's (MRC IEU's) repository of publicly available GWASs (Lyon et al., 2021). These include:

1. Age at menopause as performed with UKBB samples of European ancestry: *n* = 143,819; MRC IEU ID: ukb‐b17422 (Elsworth, 2018).
2. Alzheimer's disease incidence with samples from participants of European ancestry in the International Genomics of Alzheimer's Project (IGAP; PMID: 30820047) *n* = 63,926 with 21,982 cases and 41,944 controls; MRC IEU ID: ieu‐b‐2 (Kunkle et al., 2019)
3. Family history of AD disease with UKBB samples of European ancestry: *n* = 408,942, with 53,042 cases and 355,900 controls; AD cases were extracted from the UKBB self‐report (field 20,002), International Statistical Classification of Diseases and Related Health Problems, 10th Revision (ICD‐10) diagnoses (fields 41,202 and 41,204) and ICD‐10 cause of death (fields 40,001 and 40,002) data. UKBB participants with a first‐degree relative (biological father, mother, or sibling) who had AD/dementia were included as proxy cases, and when more than one individual in a family had AD, only one index case was included; MRC IEU ID: ebi‐a‐GCST90012878 (Schwartzentruber et al., 2021)
4. Cognitive function (PMID: 35534559) with samples from participants of European ancestry across 19 cohorts; *n* = 22,593; MRC IEU ID: ieu‐b‐4838 (Howe et al., 2022).

The remaining GWAS datasets were obtained from repositories maintained by the studies' original authors. These include:

1. Healthspan (protective ratio) with samples from UKBB participants of European ancestry (PMID: 30729179): *n* = 300,447 (Zenin et al., 2018)
2. Parental lifespan (protective ratio) of the LifeGen consortium (included UKBB samples of European ancestry; PMID: 30642433); *n* = up to 1,012,240 (Timmers et al., 2019)
3. Intrinsic epigenetic age acceleration (IEAA; a derivative of the Horvath clock (Horvath, 2013)) with a GWAS meta‐analysis of 28 cohorts of European ancestry (PMID: 34187551) (McCartney et al., 2021a); *n* = up to 34,461 (McCartney et al., 2021b)
4. GrimAge acceleration with a GWAS meta‐analysis of 28 cohorts of European ancestry (PMID: 34187551) (McCartney et al., 2021a): *n* = up to 34,467; (McCartney et al., 2021b)
5. Age at menopause of the ReproGen consortium with samples of European ancestry (PMID: 34349265) *n* = up to 201,323 (Ruth et al., 2021b)

The genetic correlations were calculated using pre‐computed linkage disequilibrium (LD) scores for Europeans (available at: https://github.com/bulik/ldsc/wiki/Heritability‐and‐Genetic‐Correlation#ld‐scores) using LD score regression (ldsc v1.0.1) (Bulik‐Sullivan et al., 2015), which estimates the shared genetic effects between two traits. The results of the genetic correlations are displayed in a forest plot created R 4.1.3 with the ggforestplot package (Scheinin et al., 2021). A Bonferroni‐threshold (*p*‐value <0.05/15 = 0.003) was set for the genetic correlations to account for multiple testing. The code for running ldsc is publicly available: https://github.com/bulik/ldsc/.

### 2.4 RNA‐seq analysis

We analyzed transcriptomic data from the ROS and MAP cohorts (Bennett et al., 2018a). The normalized count matrix from dorsolateral prefrontal cortex samples was obtained from publicly available data (synapse ID: syn3388564). We evaluated differentially expressed genes using DESeq2 (Love et al., 2014) version 1.34.0, which relies on a negative binomial generalized linear model. First, we calculated genes associated with continuous AAM (GAMs) in surgical and natural menopause groups separately, adjusting for the potential confounders from the cognition score analysis, in addition to age at death and the technical covariates, RNA integrity and postmortem interval.

Next, we calculated enrichment of these GAMs for the 50 Hallmark Pathways (Liberzon et al., 2015) using fast Gene Set Enrichment Analysis (fGSEA) (Korotkevich et al., 2016) to gain insight in which molecular processes were related to age at menopause. We also used fGSEA to check for enrichment of cognition related genes from the Mount Sinai Brain Bank (MSBB), an independent cohort. This gene lists were calculated as follows. We chose the two brain regions in the frontal cortex available from the MSBB dataset, namely Broadman Area (BA) 10 and BA 44. For each BA, we calculated genes whose expression correlated with Clinical Dementia Rating (CDR), the cognition measure available for MSBB participants. The CDR evaluates cognitive, behavioral, and functional aspects of Alzheimer disease and other dementias. We included technical covariates (postmortem interval, RNA integrity number, and sequencing batch) in the association to prevent technical noise from impacting the gene sets. We then chose the top 500 genes by *p*‐value that were positively correlated with cognition (COG‐POS) and the top 500 genes by *p*‐value that were negatively correlated with cognition (COG‐NEG) in each region to generate four gene sets (two regions with POS and NEG for each). To compare genes found in natural menopause only and surgical menopause only populations, we examined genes in the leading edge (Subramanian et al., 2005) of each enrichment analysis as implemented by fGSEA. We compared the overlap of the two leading edge gene lists using a hypergeometric (phyper, base R) test in R 4.1.

### 2.5 Metabolomics data analysis

The concentration of 41 bile acids in postmortem brain samples from ROSMAP were measured at the University of Hawaii cancer center using ultra‐performance liquid chromatography coupled to a tandem mass spectrometry, and metabolites with concentrations below the limit of detection were imputed using half of the limit of detection for each bile acid as previously described (MahmoudianDehkordi et al., 2019). We relied on previous studies (MahmoudianDehkordi et al., 2019) to select bile acid ratios of interest: chenodeoxycholic acid: cholic acid (CDCA:CA) for measuring the activation of the alternate pathway, deoxycholic acid (DCA):CA, lithocholic acid (LCA):CDCA, and ursodeoxycholic acid (UDCA): CDCA to measure conversion of gut microbiome to secondary acids. Generalized linear models with a log‐link function was used to test for association between AAM and bile acid concentration, including the same covariates as used in the cognition analyses, plus age at death. False discovery rate (FDR) corrections were applied to the *p*‐values of the association between AAM and bile acid concentrations.

Concentrations of 1055 metabolites were calculated using the Metabolon Precision Metabolomics platform as previously described in postmortem brain samples from ROSMAP (Novotny et al., 2023). For each menopause type, we excluded metabolites that were missing in half or more samples. Generalized linear models with a log‐link function was used to test for association between AAM and metabolite concentration, including the same covariates as used in the cognition analyses, plus age at death. Analyses were done separately in natural menopause and surgical menopause. FDR corrections were applied to the *p*‐values of the association between AAM and metabolite concentration.

### 2.6 miRNA analyses

microRNA (miRNA) from women undergoing oophorectomy from a previously published dataset (Baloun et al., 2022) were reanalyzed to understand which miRNA's abundance changes significantly after surgical menopause. DESeq was used to compare miRNA expression abundance from matched pre‐ and post‐menopause samples from 11 women not on hormone replacement therapy. We included a covariate of sample ID in the regression since the data were matched, as per the DESeq2 tutorial. We then predicted the target mRNA species of the significantly differentially expressed miRNA, and looked at pathway enrichment of said targets using miRPathDB 2.0 (Kehl et al., 2020), relying on the intersection of the predicted targets from different miRNA databases available within miRPathDB 2.0. We relied on gene ontology: molecular function (GO:MF) pathways for enrichment analyses within miRPathDB 2.0.

## 3 RESULTS

### 3.1 AAM is associated with cognitive function at ages 70–90

**FIGURE 1:**
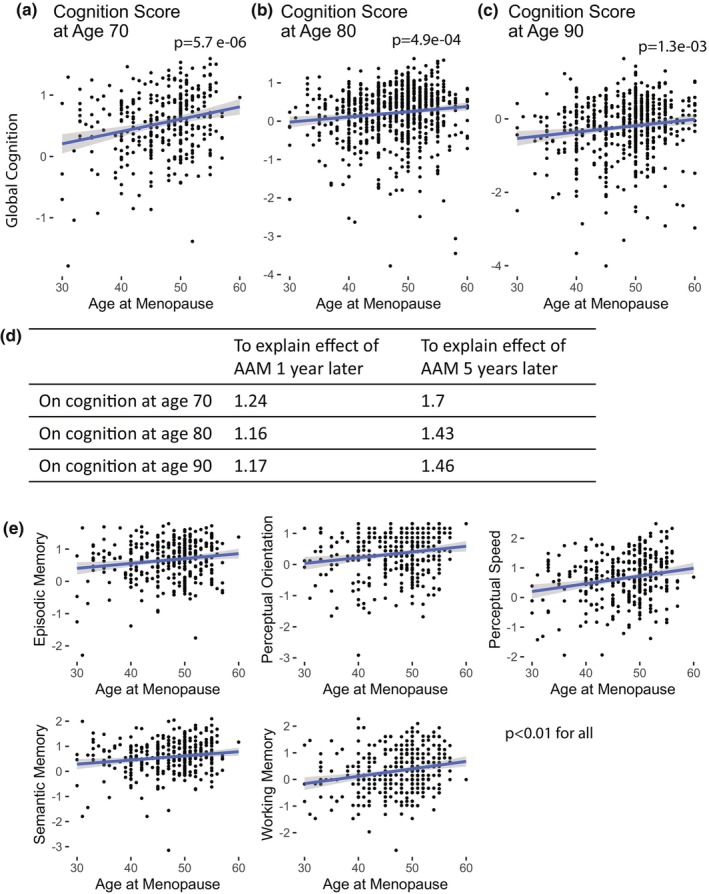
Age at menopause and cognitive function in the elderly. (a–c) Age at menopause (AAM) and cognition scores at ages 70, 80, and 90 (d) E‐value analysis shows the effect size needed to completely explain the relationship between AAM and cognition at ages 70, 80, and 90. Effect sizes are shown for a 1‐year difference in AAM and a five‐year difference in AAM. (e) Cognitive function subdomain analysis shows all five subdomains are associated with AAM. See Table S5 for full results.

We investigated the relationship between AAM and cognition scores at ages 70, 80, and 90 (*N* = 332 at 70, *N* = 807 at 80, and *N* = 545 at 90). We performed a combined analysis on ROS and MAP cohorts (Exclusion criteria in Figure S1), which were designed to have consistent processing and data collection, and were similar on a variety of demographic measures (Table S2). The mean AAM in these cohorts is consistent with population level means (Figure S2) (Davis et al., 2015). AAM was significantly associated with global cognition scores across all three decades (Figure 1a–c; Table S3). This remained after adjusting for a priori selected potential confounders thought to be associated with cognition or AAM, extending those previously tested (Bove et al., 2014). E‐value (VanderWeele & Ding, 2017) analysis suggests an unmeasured confounder is unlikely to be completely responsible for the observed association (Figure 1d). Separate analyses of those with surgical menopause and natural menopause were each consistent with the combined analysis (Table S4). We then focused on cognition at age 70 to perform various sensitivity analyses, restricting our sample to examine ROS participants only, MAP participants only, and a subgroup of consistently hormone replacement therapy (HRT)‐negative subjects—all findings were consistent with our primary analysis (Table S4). Additionally, analysis of each of the five cognitive subdomains (episodic memory, perceptual orientation, perceptual speed, semantic memory, and working memory) confirmed that the association of cognition score and AAM holds for all subdomains measured (Figure 1e, Table S5).

We analyzed cognition trends over time by using a linear mixed model (see Section 2) to account for repeated measures in the same individual. We examined if cognitive decline after age 65 was associated with AAM and the previously mentioned potential confounders. In natural menopause only, we found that those with later AAM started at a higher cognitive score baseline at age 65 (*p* = 0.04, Table S6) and although not significant, we saw a trend among those with surgical menopause for those with later AAM having a less steep slope of decline (*p* = 0.11, Table S6). This suggests that although cognition is associated with AAM across groups, the underlying mechanisms may be different.

### 3.2 AAM is inversely associated with accelerated prefrontal cortex aging

**FIGURE 2:**
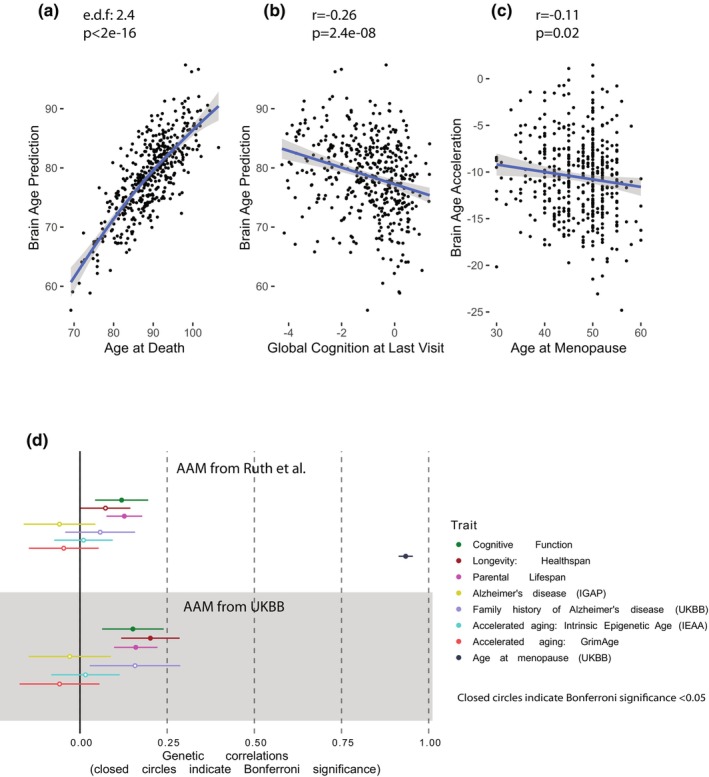
Genetic and epigenetic associations with AAM. (a) Biological age in the brain is associated with chronological age (at death) and (b) inversely correlated with cognition at last visit (c) Brain age acceleration (biological age—chronological age) is correlated with age at menopause. Full results from regression with covariates in Table S7. (d) Genetic correlations of AAM from two GWAS cohorts with 7 cognition‐ and aging‐related traits. Full results in Table S8.

We then investigated if there is an association between AAM and biological age in the brain, which to our knowledge has never been tested before. We calculated brain age using CorticalClock, a DNA methylation clock specifically trained to have higher accuracy for cortical samples (Shireby et al., 2020). Biological age, measured with the CorticalClock applied to postmortem prefrontal cortex samples, tracked with chronological age, via a slightly nonlinear positive relationship (estimated degrees of freedom 2.4, *p*‐value <2e‐16, *N* = 431, Figure 2a). Biological age was inversely correlated with cognition score at the last visit before death (*r* = −0.26, *p* = 2.4e‐08, *N* = 431, Figure 2b) as expected, confirming that the clock is reliable and meaningful for these samples. We calculated age acceleration as biological age—chronological age, and found higher age acceleration was correlated with earlier age at menopause (*r* = −0.11, *p* = 0.02, *N* = 431, Figure 2c). To more robustly examine this relationship, we regressed AAM and age at death (chronological age) from biological age, including the same covariates as the cognition analyses (see Section 2). The association between predicted brain age and AAM remained significant (*N* = 431, *p* = 0.02, Table S7), and the coefficient was similar across natural only (−0.072), surgical only (−0.075), and combined (−0.077) menopause groups. This indicates that AAM is predictive of age acceleration in the prefrontal cortex up to four decades later, and that this may be related to cognitive changes, given the correlation between brain age and cognitive function.

### 3.3 AAM is genetically correlated with longevity and cognitive function

We next wanted to understand the different contributing factors—whether genetic or epigenetic— that underlie the phenotypic correlations observed: AAM and cognition, and AAM and brain aging. We started with genetic factors and utilized genetic correlations to measure shared genetic drivers between the traits of interest, relying on two cohorts of GWAS studies for AAM (Elsworth, 2018; Ruth et al., 2021b). We found a significant positive genetic correlation (rg) between AAM and cognitive function (UKBB rg = 0.15 *p* = 7.49e‐04; Ruth et al. rg = 0.12 *p* = 2.07e‐03, Figure 2d, Table S8). Additionally, AAM was significantly positively correlated with longevity measures: parental lifespan in both cohorts (UKBB rg = 0.16 *p* = 4.28e‐07; Ruth et al. rg = 0.13 *p* = 1.1e‐06) and healthspan, measured as disease‐free survival, in UKBB (rg = 0.20 *p* = 2.20e‐06). We found a consistent but nonsignificant trend in the Ruth et al. cohort (Ruth et al., 2021b) for healthspan (rg = 0.07 *p* = 0.04). Neither AAM cohort displayed a genetic correlation between AAM and AD, replicating previous work (Ruth et al., 2021a). We found no association between AAM and AD pathology (global pathology, amyloid plaques, or tau tangles) at the phenotypic level, either (Figure S3). Although the genetic correlations between AAM and longevity were significant, they were modest in size (rg < =0.2). Additionally, we did not see evidence of genetic correlation between AAM and accelerated aging, despite there being a phenotypic correlation (Levine et al., 2016), suggesting that the environment plays a significant role, and that epigenetic mechanisms likely link AAM to brain aging and cognition. Previous work that identified a phenotypic correlation between AAM and accelerated age found it in blood only, and not in saliva or buccal epithelium (Levine et al., 2016), indicating that there may be tissue‐specific environmental effects. We therefore turned to transcriptomic data from postmortem brain samples to gain additional information about the mechanisms connecting AAM and cognition/aging.

### 3.4 Cognition‐related gene expression is enriched among genes associated with AAM in prefrontal cortex

We relied on postmortem prefrontal cortex samples in the ROS and MAP cohorts to calculate genes associated with AAM (GAMs). We corrected for aforementioned confounders, in addition to technical covariates relevant for RNA‐seq (*N* = 373, see Section 2). We examined individuals with natural menopause separately (*N* = 264) from those with surgical menopause (*N* = 109) to understand which patterns were shared, and which were different, in light of the differences in cognition over time analyses (Table S6).

To understand transcriptomic changes that might be relevant for cognition, we derived cognition gene sets from an independent cohort, the Mount Sinai Brain Bank (MSBB). We calculated these gene sets separately in each of two subregions of frontal cortex, Broadman area (BA) 10 and BA44, to increase confidence in our findings. Both subregions are in the frontal cortex, maintaining their relevance to the gene expression from frontal cortex from ROSMAP. In addition to bulk brain transcriptomics, the MSBB cohort also tracked cognition of cohort participants, thus allowing us to determine gene expression patterns that correlate with cognition. We define COG‐POS as the top 500 positively correlated genes with cognition, ranked by *p*‐value, and COG‐NEG as the top 500 negatively correlated genes (see Methods) in each BA.

**FIGURE 3:**
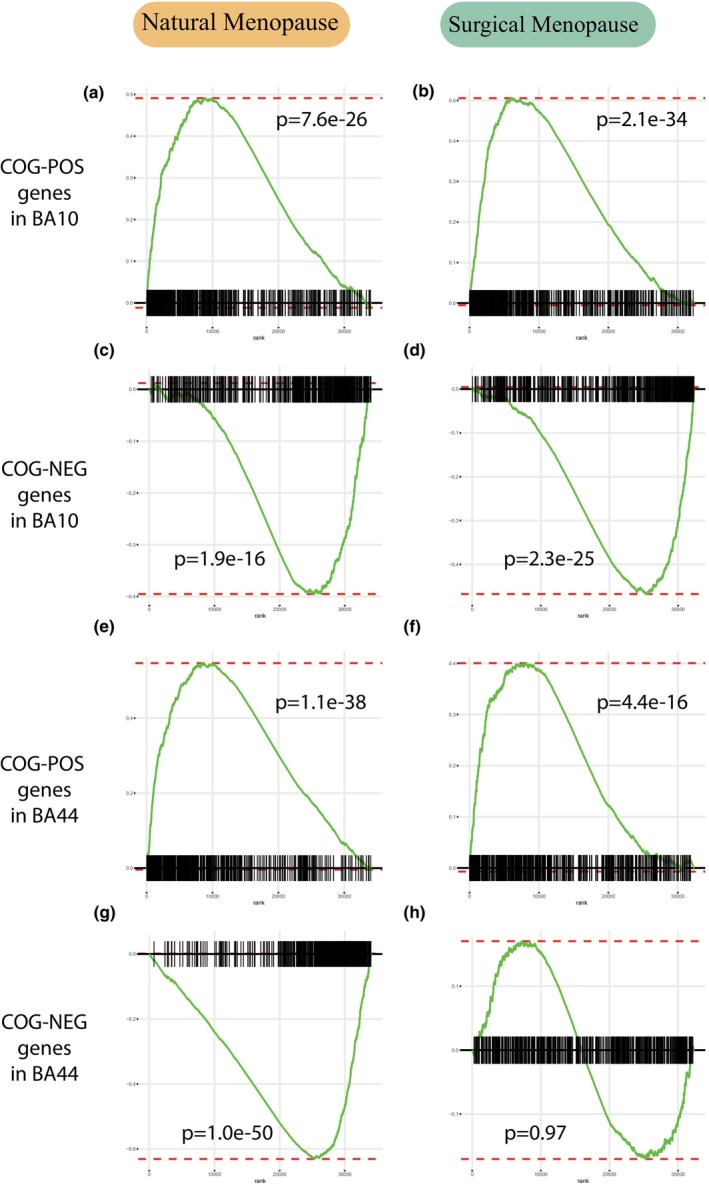
Genes associated with cognition are enriched for genes associated with AAM in directions concurrent with phenotypic findings. (a, b) There is significant enrichment of genes positively associated with cognition (COG‐POS) in Broadman Area (BA) 10 among genes positively associated with AAM in both natural and surgical cohorts (c, d) There is significant enrichment of genes negatively associated with cognition (COG‐NEG) in BA 10 among genes negatively associated with AAM in both natural and surgical cohorts. (e, f) The same analysis as in (a, b) but performed using COG‐POS gene set derived from BA 44, another area within frontal cortex. The same analysis as in (c, d) but performed using COG‐NEG gene set derived from BA 44.

In both surgical and natural menopause cohorts, we found a positive enrichment of BA10 COG‐POS genes among GAMs (Figure 3a,b) using fast Gene Set Enrichment Analysis (fGSEA) (Korotkevich et al., 2016), which detects enrichments of gene sets based on rankings. Similarly, we found a significant negative enrichment of BA10 COG‐NEG genes (Figure 3c,d; *p* < 0.001) among GAMs. This means that genes associated with better cognition have increased expression with increasing AAM, and genes associated with poorer cognition have decreased expression with increasing AAM. The COG‐POS and COG‐NEG gene lists from BA44 confirmed these findings (Figure 3e–h). Together, these results further support that early menopause, whether surgical or natural, is associated with poor cognition.

### 3.5 Age at menopause is associated with changes in bile acid metabolism

**FIGURE 4:**
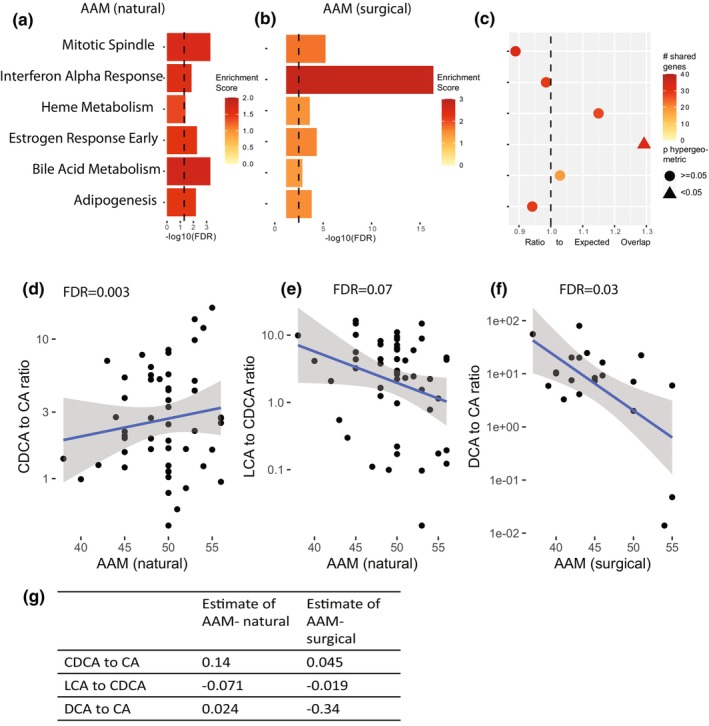
Pathways associated with AAM in both natural and surgical menopause, in the same direction. (a, b) Enrichment of Hallmark pathways that are associated with AAM in both natural and surgical groups. (c) The significance of overlap of leading edge gene sets between surgical and natural menopause in each pathway, based on the hypergeometric test. The response to estrogen has a higher overlap in leading edge gene sets by type of menopause than expected by chance. The rest of the pathways have an overlap no more than that expected by chance. Row names (pathways) are preserved across (a)–(c). (d–f) Ratios of bile acid concentrations that are significantly associated with age at natural menopause (d, e) and age at surgical menopause (f). (g) Comparison of effect size of AAM and each bile acid ratio from the linear regression results, by menopause type. Full results in Table S9.

To understand the aging‐related changes in the brain associated with AAM, we used fGSEA to calculate enrichment among GAMs using a database of 50 well‐characterized biological pathways (the Hallmark gene sets (Liberzon et al., 2015)) in each type of menopause. Since the phenotypic correlation between AAM and accelerated aging was consistent across menopause type, we started with pathways whose direction of association with AAM was consistent across types. Six pathways were significantly positively associated with AAM in both natural and surgical groups (Figure 4a,b) and none were significantly negatively associated with AAM in both groups. We extracted the leading edge (Subramanian et al., 2005): a core subset of genes from each analysis present at the peak enrichment score (Figure S4, Appendix S2). We used the hypergeometric test to check for overrepresentation or underrepresentation of genes found in common among leading edges from GAMs of the surgical group and GAMs of the natural group, for each pathway (Figure 4c). Genes in the early response to estrogen pathway were enriched for shared transcriptomic signatures between groups (*p* = 0.016). This might be due to the fact that hormonal cycling ceases at menopause, regardless if surgical or natural.

Five other pathways were increased in both groups, although their overlap of leading edges was no different that by chance, suggesting that although these pathways are associated with AAM among both types, it is different member genes responsible for enrichment in each. Three out of these five shared pathways were related to metabolism: heme metabolism, bile acid metabolism, and adipogenesis. Bile acids are the products of cholesterol metabolism, primarily through two pathways: the classical, which leads to cholic acid (CA), and the alternate, which leads to production of chenodeoxycholic acid (CDCA) (Baloni et al., 2020). We turned to bile acid concentrations measured from postmortem brain tissue to further investigate this finding (natural *N* = 54, surgical *N* = 20). Ratios of bile acids can indicate changes in enzymatic activities of the liver and the gut, we tested four ratios from previously published work (MahmoudianDehkordi et al., 2019) for their association with AAM (Figure 4d–g, Table S9). Later AAM was associated with an increased CDCA:CA ratio by linear regression in natural menopause (FDR = 0.003; Figure 4d; Table S9) but not in surgical menopause (FDR >0.5, Figure S5). CDCA:CA measures activation of the alternate pathway of cholesterol metabolism (MahmoudianDehkordi et al., 2019). One of the key genes in the alternate pathway, CYP27A1 (Grant & DeMorrow, 2020), was found in the leading edge of GAMs of natural menopause, but not surgical menopause, showing further alignment between transcriptomic and metabolomic data.

Additionally, in those with natural menopause, lithocholic acid (LCA): CDCA was associated with AAM (FDR = 0.07; Figure 4e, Table S9). LCA is a secondary acid derived from CDCA. Production of LCA by gut microbiota is also part of the alternate pathway of cholesterol metabolism (Grant & DeMorrow, 2020). Among those with surgical menopause only, the deoxycholic acid (DCA): CA ratio was inversely associated with AAM (FDR = 0.03; Figure 4f; Figure S6). DCA is another secondary acid derived from gut microbiota, and suggests that the gut plays a role in both types of menopause. These orthogonal observations confirm and refine the transcriptomic finding that later age at menopause is associated with altered bile acid metabolism, albeit through bile acid ratios and expression of different metabolism genes in each menopause type.

### 3.6 Age at natural menopause is associated with transcriptional changes in canonical aging pathways

**FIGURE 5:**
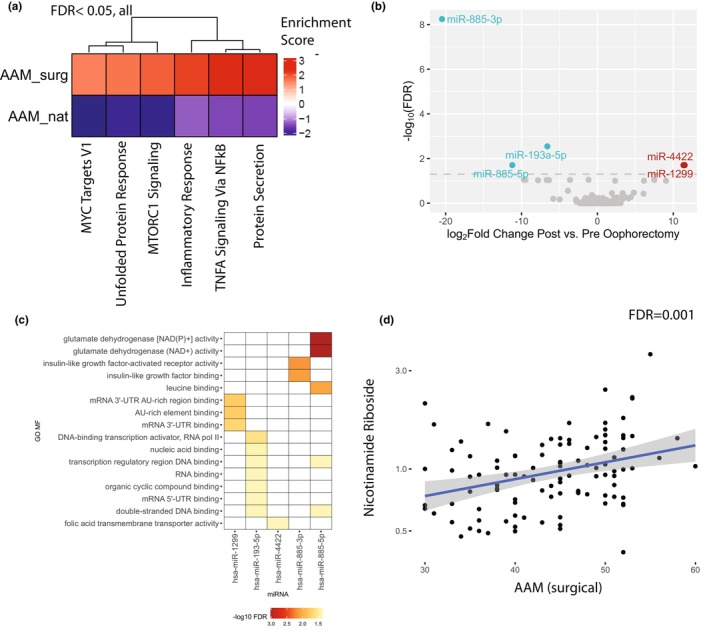
Molecular mechanisms that connect AAM to aging. (a) Hallmark pathways that are significantly associated with AAM in opposite directions in natural and surgical groups. (b) miRNA that have significant expression changes between matched pre‐ and post‐oophorectomy samples from the same women (c) Pathway enrichment of experimentally validated targets of miRNA significantly perturbed after oophorectomy. (d) The concentration in the brain of nicotinamide riboside, one of the key NAD+ precursors, is associated with age at surgical menopause.

Surgical menopause is often performed in individuals at higher risk of ovarian cancer or to relieve pain from endometriosis. Given these underlying factors, and the differences observed in cognition trajectories and leading edge gene sets of shared pathways, we next examined pathways whose direction of association with AAM is opposite between surgical and natural menopause groups. We found that among those with natural menopause, AAM is negatively associated with expression of genes in pathways relating to inflammation, unfolded protein response, and mammalian target of rapamycin complex 1 (mTORC1) signaling (FDR <0.05), meaning that those with later AAM have lower expression of genes in these pathways (Figure 5a). This could explain the association seen between AAM and accelerated brain aging, as inflammation and loss of protein homeostasis are hallmarks of aging (López‐Otín et al., 2023). mTORC1 signaling in particular is one of the key pathways in longevity—inhibiting mTORC increases lifespan in all model organisms studied (Weichhart, 2018), and so lower mTORC signaling among those with later AAM is consistent with our prefrontal cortex aging findings. Notably, the association between these pathways and AAM is reversed in surgical menopause, which suggests there must be alternate mechanisms in surgical menopause that relate to aging.

### 3.7 Surgical menopause is associated with key aging‐related metabolites

Risk factors for conditions such as cancer and endometriosis may lead to earlier timing of surgical menopause. To disentangle the effects of surgical menopause from the effects of conditions associated with surgical menopause, we turned to miRNA from matched blood samples from living women before and after oophorectomy (*N* = 24). We examined changes in circulating miRNA after surgery compared to before surgery by reanalyzing a previously published dataset (Baloun et al., 2022). Five miRNAs out of 1003 were significantly differentially expressed after oophorectomy (Figure 5b, |log2 fold change| >5 and FDR <0.05). We next looked at Gene Ontology Molecular Function (GO MF) terms of target‐mRNA species of each of these five miRNAs, relying on the miRNA targets with experimental evidence (Kehl et al., 2020). Pathway enrichment of the target genes showed the strongest enrichment for nicotinamide adenine dinucleotide (NAD+) activity (Figure 5c), suggesting that surgical menopause is associated with changes in NAD+ activity. Insulin‐like growth factor (IGF) binding genes were also predicted to be disrupted by surgical menopause.

To validate the association of metabolomic changes associated with surgical menopause, and to understand its relevancy to the brain, we relied on previously generated metabolon data (natural *N* = 230, surgical *N* = 107) for over 1000 compounds in postmortem human brain tissue in the ROS and MAP cohorts (Novotny et al., 2023). In an unbiased approach examining all metabolites, and after correcting for previously listed covariates and multiple hypothesis testing (see Section 2), we found 22 compounds significantly associated with age at surgical menopause (Table S10). All of these were unique to surgical menopause (Table S10). The NAD+ precursor nicotinamide riboside (NR) was among the top‐ranked compounds (by *p*‐value) associated with age at surgical menopause (Figure 5d, Figure S7, Table S10). NR is part of a group of seven highly correlated compounds associated with age at surgical menopause (Figure S8), mostly amino‐acid derivatives. This could be related to the finding from transcriptomics that later age at surgical menopause is associated with increased protein secretion (Figure 5a), as both could be a response to increased abundance of amino acids in the brain. The metabolomics results concur with the miRNA results that NAD+ activity is affected by surgical menopause. NR and NAD+ might contribute to the relationship between AAM and aging in this group, given the interest of NR in the aging field (Sun et al., 2021).

## 4 DISCUSSION

Overall, our data show robust evidence that AAM is predictive of brain aging at the molecular and cognitive levels. In both surgical and natural menopause, earlier AAM is associated with accelerated brain aging and lower cognition scores, albeit via different trajectories. Gene expression corroborated the association between AAM and cognition. To understand what mediates this association, we separately analyzed the populations with natural menopause and surgical menopause. In both, we saw signatures of response to estrogen increased with later AAM, suggesting that the timing of hormonal signaling cessation has a lasting impact on the brain. Bile acid metabolism genes were associated with AAM in both groups, but the leading edge of gene sets was distinct. This was validated by different bile acid ratios being associated with AAM in natural (primarily ratios in the alternate pathway) and surgical (primarily ratios in the classical pathway) menopause groups. In both groups, secondary acid: primary acid ratios (DCA: CA, LCA: CDCA) were associated with AAM. Secondary acids are known to be produced by gut microbiota (MahmoudianDehkordi et al., 2019), suggesting that the involvement of the gut as a modulator between reproductive aging and brain aging. The use of animal models may elucidate in finer detail how secondary bile acids respond to reproductive changes, and cell culture can be used to model how they go on to affect brain aging.

There is growing evidence that circulating miRNA can affect gene expression in distal tissues (Wu et al., 2023) and that circulating miRNA are perturbed in neurodegeneration (Grasso et al., 2014). We turned to matched miRNA from pre‐ and post‐oophorectomy from the same human donors for additional insight. Post‐oophorectomy, miR‐885‐3p expression decreased in the blood, and this miRNA is known to have targets enriched in IGF binding genes. Tissue‐specific genetic knockout experiments in the endoderm (the gut) of *C*. *elegans* causally implicate inhibition of IGF gene family in the relationship between lifespan and depletion of germ cell precursors, whereby depletion of germ cells no longer increases lifespan in mutants with disrupted IGF signaling (Hsin & Kenyon, 1999; Mason et al., 2022). This suggests that IGF is a conserved link between reproductive organs and aging. miR‐885‐5p expression also decreased post‐oophorectomy, and its targets are enriched for NAD+ activity. NAD+ has emerged as a key mediator between caloric restriction and healthspan. Nicotinamide riboside, whose concentration in the brain we found to be associated with age at surgical menopause, is one of the NAD+ precursors whose supplementation leads to increased NAD+ availability (Martens et al., 2018) and attenuated age‐associated metabolic changes in hematopoietic stem cells (Sun et al., 2021). Together, these findings provide additional evidence for the role of the gut in surgical menopause, and future work should evaluate if miRNA from circulation directly impacts gene expression in the brain.

We observed positive genetic correlations between AAM and cognitive function, as well as longevity measures, which potentially supports the use of AAM as a biomarker of aging. Genetic correlations are agnostic to direction. We gained further confidence in the relationship between ovarian aging and brain aging through RNA‐seq analyses, where people with earlier natural AAM showed increased expression of genes in canonical patterns of aging (a result not seen with age at surgical menopause). In particular, we saw that people with earlier natural menopause have higher mTORC1 signaling in their brain than those with later natural menopause, a pathway whose expression increases with aging in human ovaries (Jin et al., 2022). Earlier age at natural menopause may be a symptom of faster aging throughout the body, rather than a cause. Together, these multiple lines of evidence support the idea that earlier AAM is both a result of and a predictor of aging. Future work should evaluate if other markers of ovarian reserve, including measures of anti‐Müllerian hormone, estradiol, follicle stimulating hormone, and inhibin B (Ulrich & Marsh, 2019), provide a more granular understanding of reproductive age, and add additional insight to the connection to the brain.

There are several strengths of this study. This is the first study to profile molecular changes in DNA, RNA, and metabolomics in the brain by age at and type of menopause. The sample sizes are large: 2086 with epidemiological data, and 143,819 with GWAS data from the UKBB, 373 samples with RNA‐seq, 431 with DNA methylation, and 338 with metabolomics. Across various modalities of data, a consistent theme emerged: earlier age at menopause predicts accelerated brain aging. Our results find a potential correlate of cognitive decline and poor aging at a time‐point decades before the outcome is experienced, thus allowing clinicians and individuals to leverage existing interventions that promote resilience and reduce the risk of dementia. The timing of menopause is noninvasive and essentially free to measure. Individual women notice their own timing of menopause without having to schedule appointments or undergo extensive testing, and can take initiative independently.

We acknowledge that there are limitations of this study: both the ROS and MAP cohorts include primarily those of European descent, as does the GWAS cohorts used for genetic correlations. This may fail to capture ancestral differences in the relationship between menopause and brain aging, and may also fail to capture how societal factors such as discrimination affect this relationship. Future studies are needed with greater diversity. All tissue samples are postmortem from an aged population, which may influence results, as there may be degradation in the postmortem interval or confounding factors associated with the cause of death. To account for this, we added multiple covariates, such as age at death and duration of the postmortem interval to our analysis, but there may be remaining unknown effects. We were not able to reliably measure the effect of HRT, but by analyzing high‐confidence non‐HRT‐users, we confirm our findings are not driven by HRT. Although we corrected for numerous potential confounders such as age at menarche, alcohol consumption, education, and smoking status, it is possible that there is residual confounding responsible for the observed association due to factors such as chronic stress or early life adversity. We also cannot rule out that part of the effect observed is due to reverse causality, whereby accelerated brain aging affects the timing of menopause, possibly through an unknown mechanism, although there is currently little evidence to suggest this. Longitudinal studies with earlier age at enrollment would add clarity to the directionality of the association.

## AUTHOR CONTRIBUTIONS

F.G.‐R. and C.M. designed the study. Statistical analyses were performed by F.G.‐R. with guidance from Y.Z. and C.M. Genetic correlations were performed by C.A. RNA‐seq analyses were performed by F.G.‐R. with guidance from R.M.R. and E.A.L. Other omics analyses were performed by F.G.‐R. F.G‐R. wrote the manuscript with major edits from E.A.L. and C.M., and feedback from all authors.

## CONFLICT OF INTEREST STATEMENT

The authors declare no conflict of interest.

## Supporting information


Appendix S1.

Appendix S2.

## Data Availability

A summary of data sources can be found in Table S11. Part of the data that support the findings of this study are openly available in GSE194086 at https://www.ncbi.nlm.nih.gov/geo/ (miRNA) (Baloun et al., 2022). Detailed instructions for accessing publicly available GWAS summary statistics for each trait are in Methods with all citations re‐listed here (Elsworth, 2018; Kunkle et al., 2019; McCartney et al., 2021b; Ruth et al., 2021b; Schwartzentruber et al., 2021; Timmers et al., 2019; Zenin et al., 2018). The ROS and MAP cohort data are not publicly available due to privacy or ethical restrictions. ROSMAP resources (Bennett et al., 2018a) can be requested at https://www.radc.rush.edu and www.synpase.org.
