# Supplementary material for: Multiomic profiling reveals timing of menopause predicts prefrontal cortex aging and cognitive function
Source: Aging Cell. 2024 Nov 5;24(2):e14395. doi: 10.1111/acel.14395 (PMC11822667; doi:10.1111/acel.14395)
Supplement: Supplementary file 1 — Appendix S1. [file ACEL-24-e14395-s001.pdf]

Figure S1. Flow Chart of Participants in ROS, MAP cohorts.

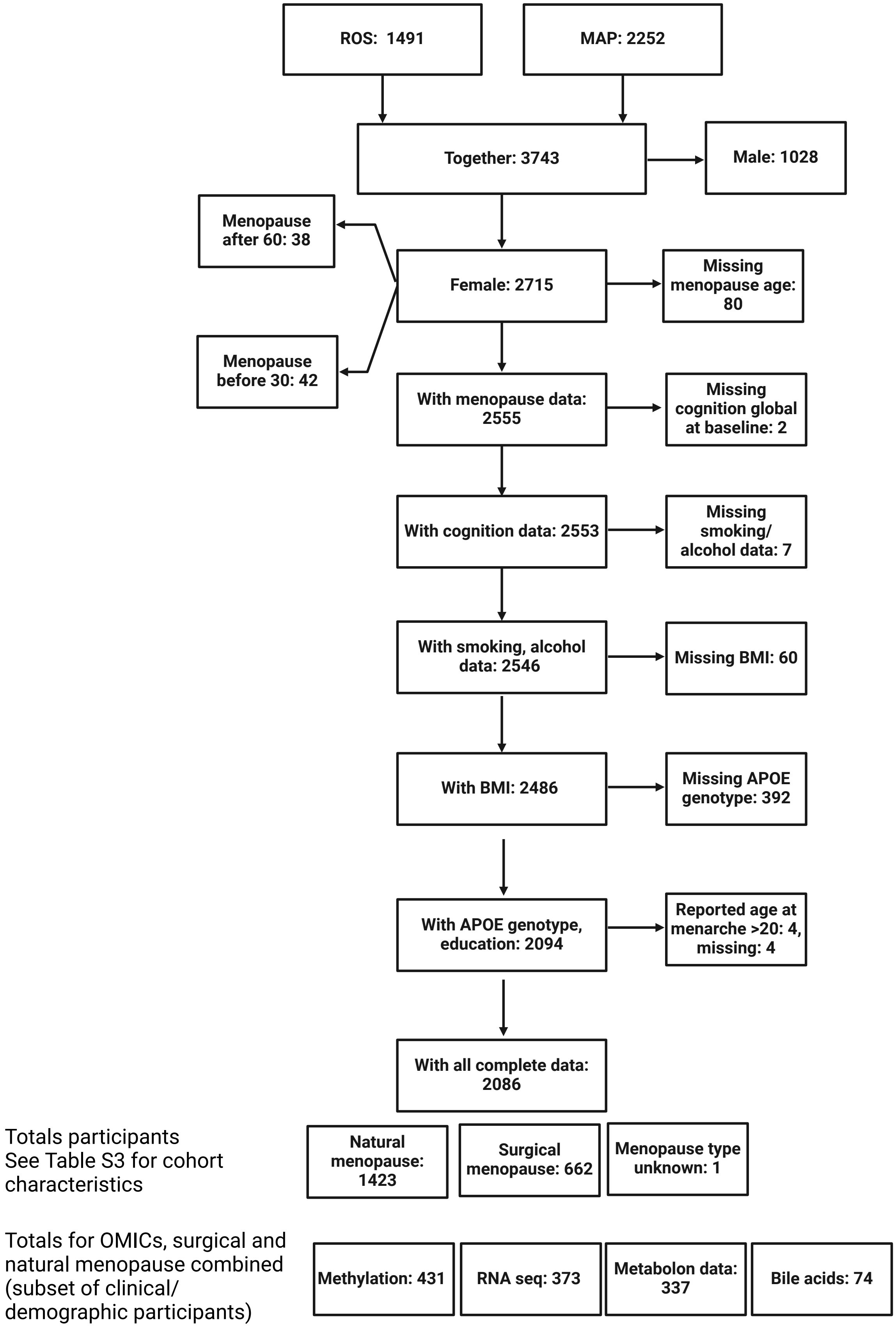

Figure S2. Age at menopause distribution among ROS/ MAP samples, separated by type of menopause.

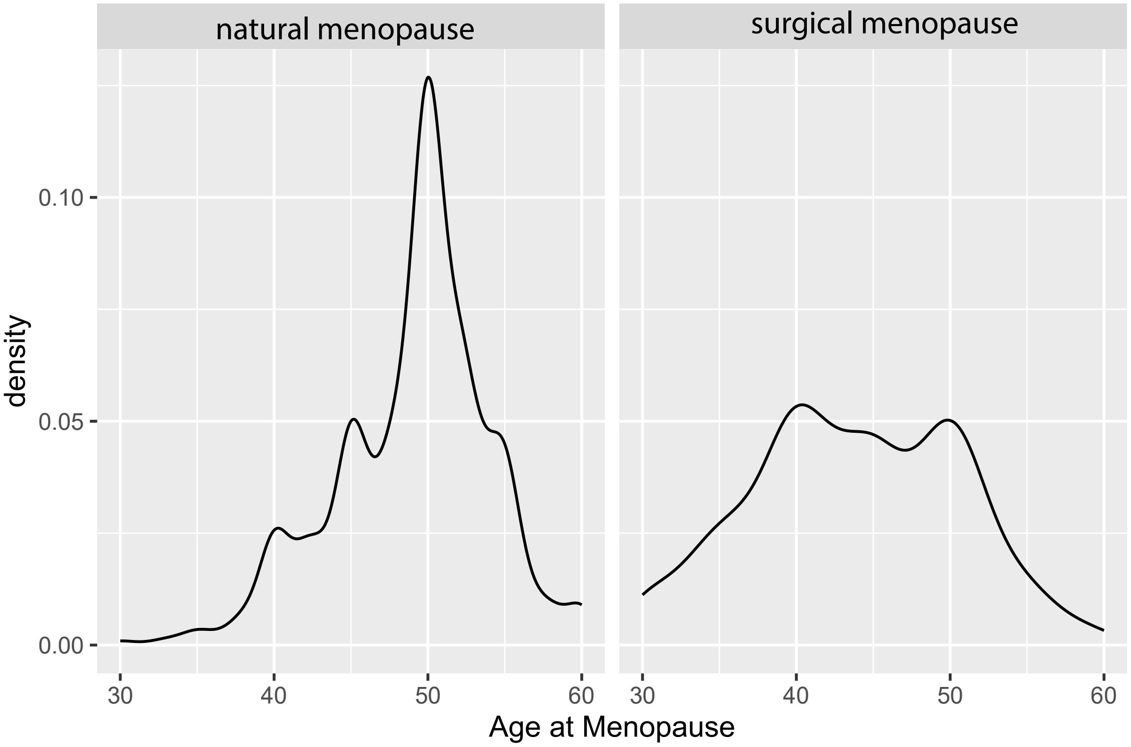

Percentile	Natural AAM	Surgical AAM
25%	46	39
50%	50	44
75%	52	50

Figure S3: No association between AAM at global pathology.
p-value > 0.1 for all

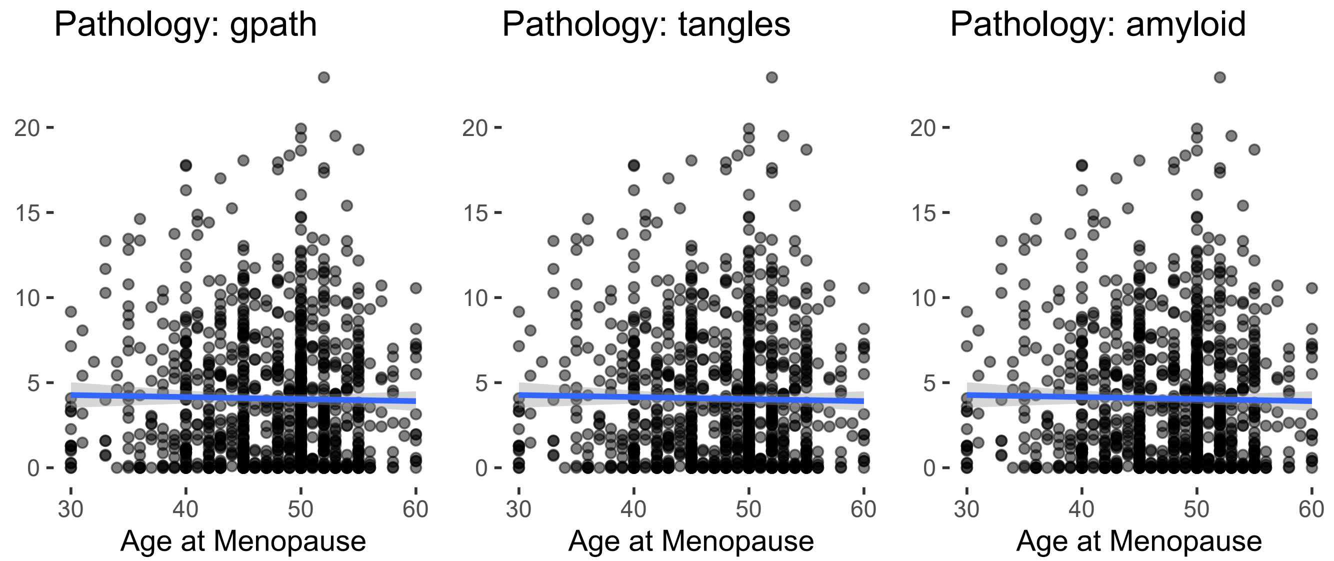

Figure S4. Leading edge example for positive encirhcment (a) and negative encirhment (b).

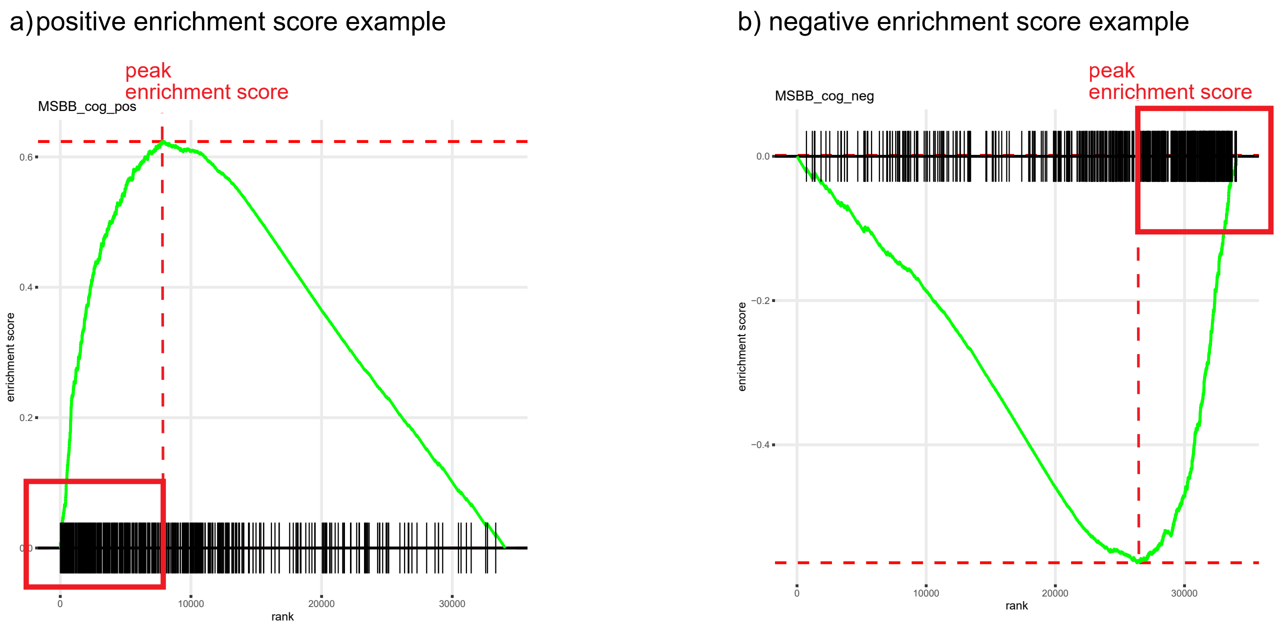

Hits inside red boxes are genes in the leading edge
See GSEA user guide for more details

Figure S5. Ratios of bile acid concentrations in the brain, by age at surgical menopause.

FDR>0.5

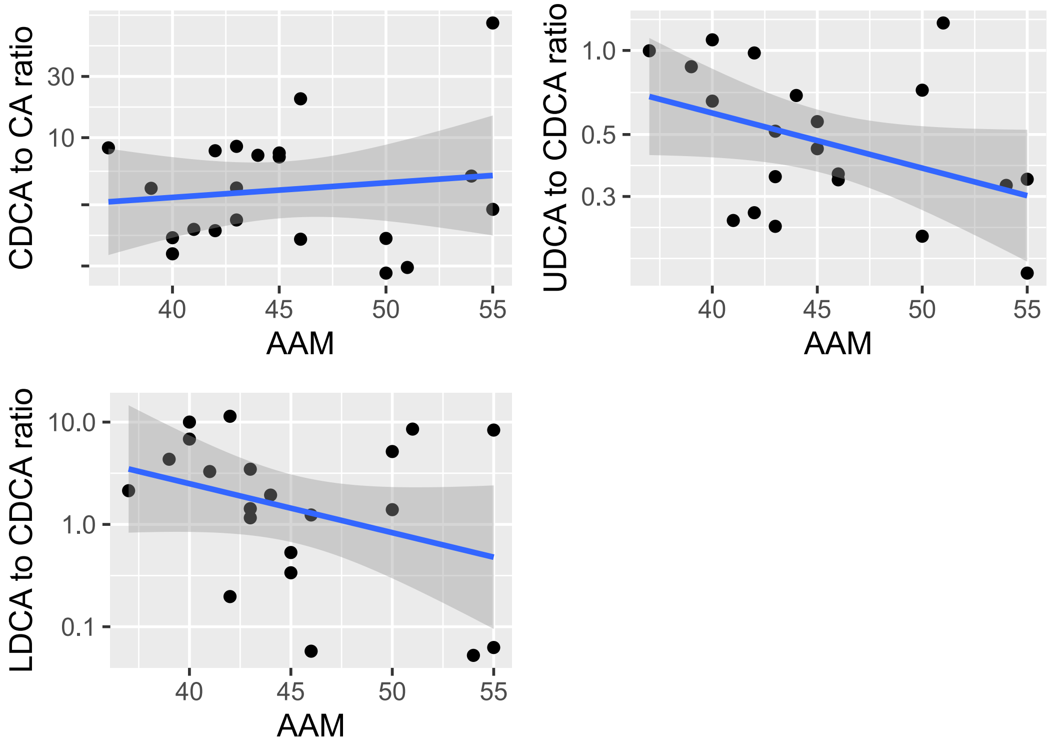

Figure S6. Ratios of bile acid concentrations in the brain, by age at natural menopause.

FDR>0.5

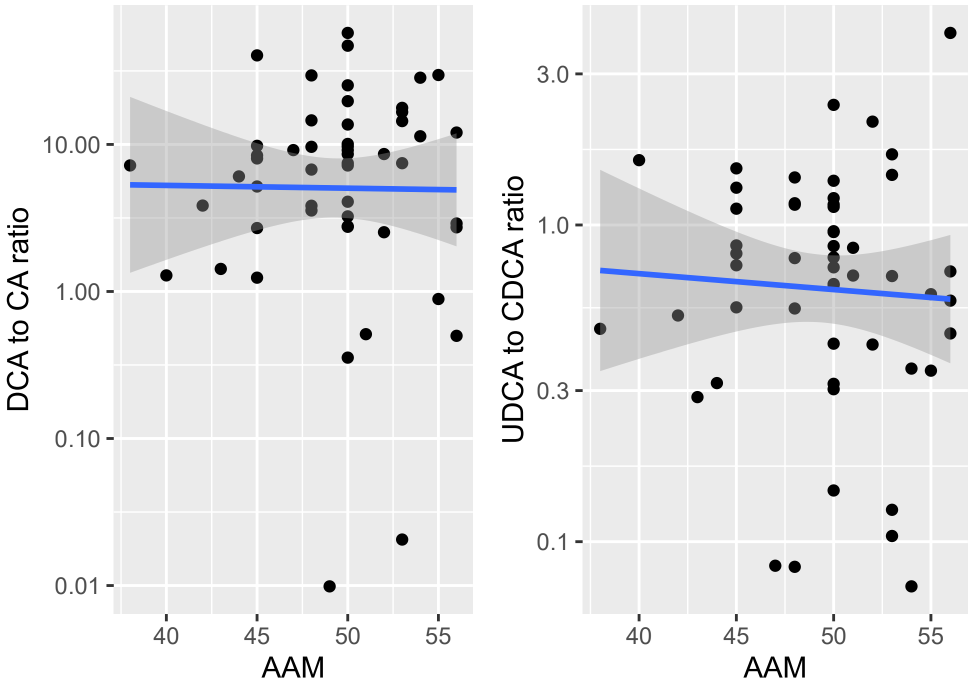

Figure S7. Nicotinamide Riboside Concentrations by Age at Menopause Among Those with Natural Menopause

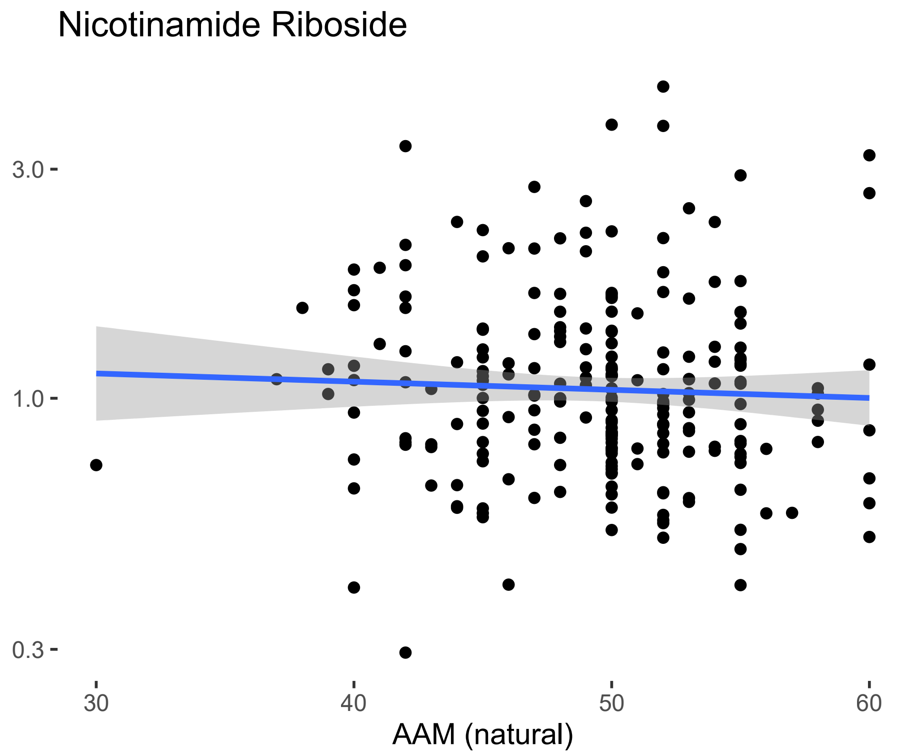

Figure S8. Correlations between metabolite concentrations that are associated with AAM in natural (top) and surgical (bottom) menopause. Statistics of metabolite associations with AAM in supplemental table 9.

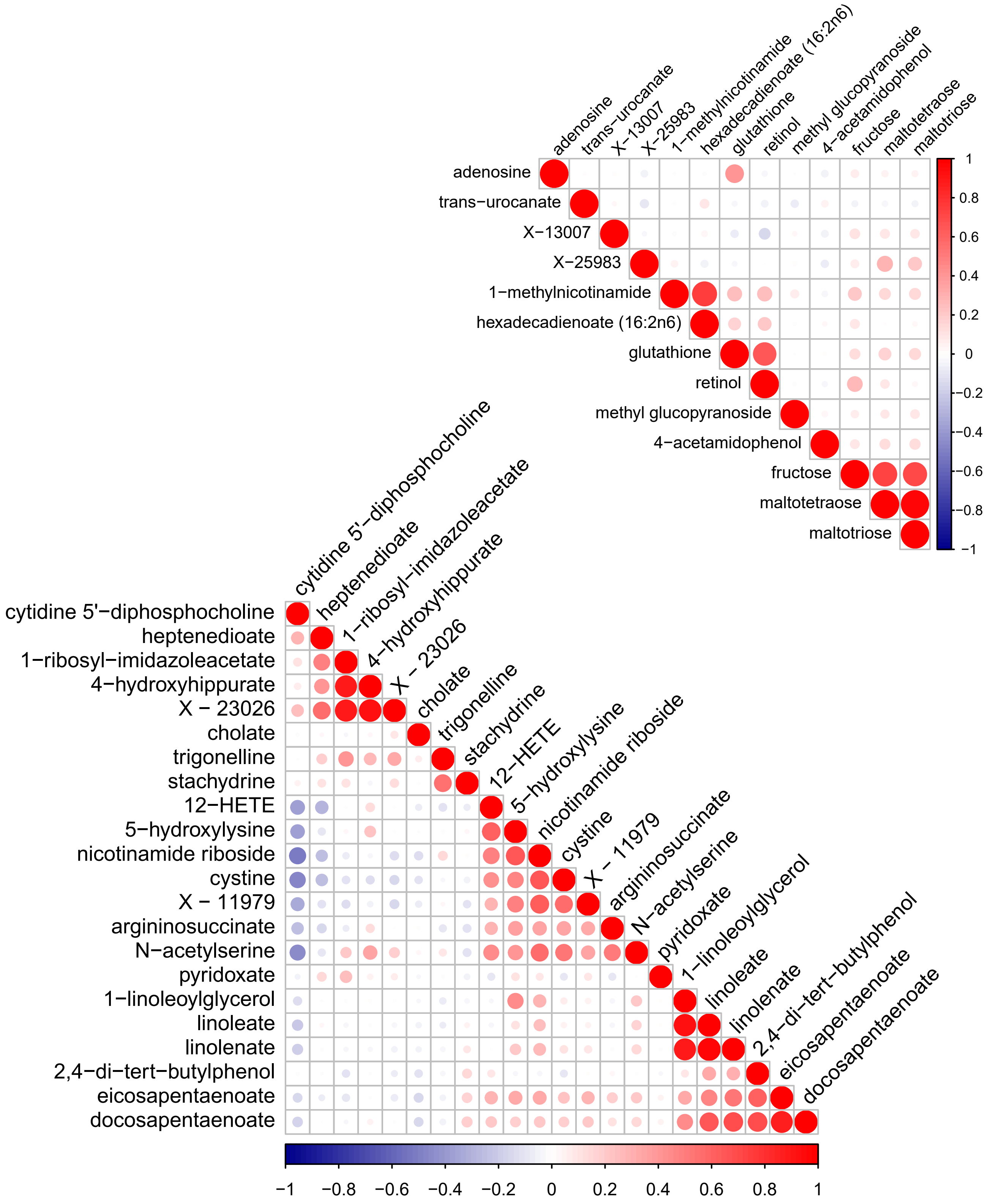

Table S1. Tests included in ROSMAP global cognition score.

test score	z-score	cognitive test	calculated domain
cts_wli	z_WLI	word list	episodic memory (cogn_ep)
cts_wlii	z_WLII	word list recall	episodic memory (cogn_ep)
cts_wliii	z_WLIII	word list recognition	episodic memory (cogn_ep)
cts_ebmt	z_EBMT	East Boston immediate recall	episodic memory (cogn_ep)
cts_ebdr	z_EBDR	East Boston delayed recall	episodic memory (cogn_ep)
cts_story	z_Story	Logical memory I (immediate)	episodic memory (cogn_ep)
cts_delay	z_Delay	Logical memory II (delayed)	episodic memory (cogn_ep)
cts_bname	z_BName	Boston naming (15 items)	semantic memory (cogn_se)
cts_catflu	z_CatFlu	category fluency	semantic memory (cogn_se)
cts_read_nart	z_read_nart	reading test - (10 items)	semantic memory (cogn_se)
cts_df	z_DF	digits forward	working memory (cogn_wo)
cts_db	z_DB	digits backward	working memory (cogn_wo)
cts_doperf	z_DOperf	digit ordering	working memory (cogn_wo)
cts_lopair	z_LOpair	line orientation	visuospatial ability/perceptual orientation (cogn_po)
cts_pmat	z_PMat	progressive matrices (16 items)	visuospatial ability/perceptual orientation (cogn_po)
cts_sdmt	z_SDMT	symbol digits modality-oral	perceptual speed (cogn_ps)
cts_nccrtd	z_NCcrtd	number comparison	perceptual speed (cogn_ps)
cts_stroop_cname	z_cname	stroop color naming	perceptual speed (cogn_ps)
cts_stroop_wread	z_wread	stroop word reading	perceptual speed (cogn_ps)

Table S2. Demographic and clinical characteristics of the ROS and MAP cohorts.

	ROS	MAP
Sample Size	856	1230
Mean age at menopause	46.9	47.7
Surgical menopause	27.4%	34.8%
Ever smoker	8.5%	38.5%
Mean alcohol consumption at peak (scaled)	0.13	0.41
Mean years of education	18.2	14.4
Average BMI	27.4	27.4

Table S3. Regression results of AAM and global cognition scores at ages 65-95.

Age at cognition measurement	Unadjusted p-value	Unadjusted estimate	Adjusted p-value	Adjusted estimate	Degrees of freedom	Number of included samples	F-statistic model
65	0.163	0.010	0.20	0.010	106	107	3.8e-05
70	6.24e-06	0.019	5.7 e-06	0.018	332	333	< 2.2e-16
75	3.73e-03	0.011	0.064	0.007	555	556	3.5e-13
80	5.14e-5	0.015	4.9e-04	0.013	807	808	< 2.2e-16
85	9.50e-03	0.010	2.5e-03	0.012	831	832	< 2.2e-16
90	0.037	0.011	1.3e-03	0.017	545	546	6.9e-6
95	0.037	0.022	0.29	0.012	151	152	0.6089

Table S4. Sensitivty analyses with subgroups for AAM and global cognition at age 70.

Subgroup	Adjusted p-value	Adjusted estimate	Degrees of freedom	Number of samples	F-statistic of model
Confirmed HRT-negative	0.01	0.018	112	113	4.5e-06
ROS only	2.4e-04	0.019	210	211	6.4e-07
MAP only	0.019	0.016	114	115	2.9e-10
Natural menopause only	0.0016	0.017	227	228	3.3e-14
Surgical menopause only	0.056	0.015	97	98	0.0014

Table S5. Regression output for AAM and cognition subdomains at age 70.

Cognitive subdomain	Adjusted p-value	Adjusted estimate	Degrees of freedom	Number of included samples	F-statistic of model
Episodic Memory	6.2e-03	0.014	332	333	1.3e-10
Perceptual Orientation	3.0e-03	0.017	332	333	2.5e-10
Perceptual Speed	1.4e-04	0.024	332	333	3.6e-11
Semantic Memory	9.4e-03	0.014	333	334	5.7e-11
Working Memory	8.2e-05	0.026	332	333	5.0e-09

Table S6. Linear mixed model for global cognition over time. Natural only (top) surgical only (bottom).

Random effects:
Formula: ~1 + age_at_visit | projid
Structure: General positive-definite, Log-Cholesky parametrization

	StdDev	Corr
(Intercept)	4.8262354	(Intr)
age_at_visit	0.0631132	-0.993
Residual	0.2677821	

Fixed effects: cogn_global ~ age_at_enrollment + AAM* age_post_65 + age at menarche + smoking + aclohol+ bmi + apoe_e4_status + educ

	Value	Standard Error	P-value
Intercept	-1.7608415	0.3952748	<0.0001
AAM	0.0124702	0.0061846	0.0440
Age at Enrollment	0.0215840	0.0025583	<0.0001
Age post 65	-0.0653810	0.0197565	0.0009
Age at Menarche	-0.0177707	0.0099601	0.0746
Education	0.0444902	0.0048848	<0.0001
Smoking (former)	0.0479512	0.0411338	0.2439
Smoking (current)	-0.3029198	0.1431152	0.0345
Alcohol	0.0380467	0.0242813	0.1174
BMI	0.0060008	0.0013869	<0.0001
APOE	-0.2031752	0.0399939	<0.0001
AAM: Age post 65	0.0000583	0.0003998	0.8842

Random effects:
Formula: ~1 + age_at_visit | projid
Structure: General positive-definite, Log-Cholesky parametrization

	StdDev	Corr
(Intercept)	5.4646597	(Intr)
age_at_visit	0.0710195	-0.994
Residual	0.2830837	

Fixed effects: cogn_global ~ age_at_enrollment + AAM* age_post_65 + age at menarche + smoking + aclohol+ bmi + apoe_e4_status + educ

	Value	Standard Error	P-value
Intercept	-1.7240353	0.4909157	0.0004
AAM	-0.0018104	0.0072047	0.8017
Age at Enrollment	0.0306564	0.0039797	<0.0001
Age post 65	-0.1010058	0.0213212	<0.0001
Age at Menarche	-0.0263765	0.0159817	0.0993
Education	0.0562588	0.0073064	<0.0001
Smoking (former)	-0.0068407	0.0616902	0.9117
Smoking (current)	0.0614586	0.1857899	0.7409
Alcohol	0.0404488	0.0366541	0.2702
BMI	0.0022400	0.0020740	0.2802
APOE	-0.2401203	0.0602881	0.0001
AAM: Age post 65	0.0007646	0.0004785	0.1101

Table S7. DNA Methylation Brain Age Regression Output

	Estimate	Standard Error	P-value
Intercept	82.6	2.7	<2e-16
Age at Menopause	-0.077	0.032	0.015
Age at Menarche	-0.12	0.13	0.35
ROS cohort	-0.038	0.51	0.94
Alcohol consumption	0.38	0.34	0.26
Former smoker (vs. never)	-0.076	0.54	0.89
Current smoker (vs. never)	-2.9	1.8	0.11
Education	0.067	0.069	0.33
BMI	0.0020	0.037	0.96
APOE e4 carrier	0.24	0.46	0.60

	Estimated degrees of freedom	F	P-value
s(Age at death)	2.33	225.4	<2e -16

R-sq.(adj) = 0.62 Deviance explained = 63%
GCV = 16.482

Table S8. Results of Genetic Correlations.

Trait 1	Trait 2	Genetic correlation: rg	Standard Error	Z	P-value
Age at menopause (Ruth et al.)	Cognitive Function	0.1195	3.88E-02	3.0806	2.07E-03
Age at menopause (Ruth et al.)	Age at menopause (UKBB)	0.934	1.04E-02	89.9406	0.00E+00
Age at menopause (Ruth et al.)	Dementia: Alzheimer's disease (IGAP)	-0.0586	5.24E-02	-1.1181	2.64E-01
Age at menopause (Ruth et al.)	Healthspan	0.0732	3.64E-02	2.0119	4.42E-02
Age at menopause (Ruth et al.)	Parental Lifespan	0.1273	2.61E-02	4.8733	1.10E-06
Age at menopause (Ruth et al.)	Accelerated aging: Intrinsic Epigenetic Age (IEAA)	0.0102	4.27E-02	0.239	8.11E-01
Age at menopause (Ruth et al.)	Accelerated aging: GrimAge	-0.0465	5.10E-02	-0.9124	3.62E-01
Age at menopause (Ruth et al.)	Dementia: family history of Alzheimer's disease (UKBB)	0.058	5.11E-02	1.1338	2.57E-01
Age at menopause (UKBB)	Age at menopause (Ruth et al.)	0.934	1.04E-02	89.9406	0.00E+00
Age at menopause (UKBB)	Cognitive Function	0.1518	4.50E-02	3.3709	7.49E-04
Age at menopause (UKBB)	Dementia: Alzheimer's disease (IGAP)	-0.0293	6.02E-02	-0.4873	6.26E-01
Age at menopause (UKBB)	Healthspan	0.202	4.27E-02	4.7339	2.20E-06
Age at menopause (UKBB)	Parental Lifespan	0.1601	3.17E-02	5.0559	4.28E-07
Age at menopause (UKBB)	Accelerated aging: Intrinsic Epigenetic Age (IEAA)	0.0159	4.99E-02	0.3178	7.51E-01
Age at menopause (UKBB)	Accelerated aging: GrimAge	-0.0584	5.84E-02	-1.0005	3.17E-01
Age at menopause (UKBB)	Dementia: family history of Alzheimer's disease (UKBB)	0.1579	6.62E-02	2.3836	1.71E-02

Table S9. Bile acid concentration ratios regression output natural (top), surgical (bottom)

Bile Acid Ratio	Estimate	Standard Error	P-value	FDR adjusted P-value	Degrees of freedom	Number of samples
CDCA to CA	0.14	0.039	0.00074	0.0029	50	51
DCA to CA	0.024	0.048	0.62	0.62	50	51
LCA to CDCA	-0.071	0.033	0.037	0.074	50	51
UDCA to CDCA	0.017	0.034	0.61	0.62	49	50

Bile Acid Ratio	Estimate	Standard Error	P-value	FDR adjusted P-value	Degrees of freedom	Number of samples
CDCA to CA	0.045	0.074	0.55	0.74	19	20
DCA to CA	-0.34	0.1	0.0078	0.031	19	20
LCA to CDCA	-0.019	0.056	0.746	0.75	19	20
UDCA to CDCA	-0.044	0.03	0.18	0.35	19	20

Table S10. Metabolite concentration regression output natural (top), surgical (bottom)

Compound Name	Estimate	Standard Error	P-value	FDR
1-methylnicotinamide	0.2062	0.0300	6.35E-11	4.69E-08
glutathione, reduced (GSH)	0.5024	0.1009	1.30E-06	1.38E-04
retinol (Vitamin A)	0.1520	0.0313	4.02E-06	4.94E-04
trans-urocanate	0.7726	0.1666	8.19E-06	8.64E-04
adenosine	-0.3401	0.0926	3.00E-04	1.84E-02
fructose	-0.0556	0.0124	1.27E-05	8.02E-08
4-acetamidophenol	-0.0731	0.0180	7.06E-05	6.52E-03
maltotetraose	-0.1121	0.0296	1.96E-04	1.33E-02
maltotriose	-0.0724	0.0204	4.79E-04	1.33E-02
methyl glucopyranoside (alpha + beta)	0.2237	0.0402	8.42E-08	1.55E-05
hexadecadienoate (16:2n6)	0.1940	0.0345	8.05E-08	1.55E-05
X-13007	-0.0390	0.0111	5.55E-04	3.15E-02
X-25983	0.0465	0.0119	1.39E-04	1.14E-02

Compound Name	Estimate	Standard Error	P-value	FDR
4-hydroxyhippurate	0.1392	0.0247	5.44E-07	4.04E-04
nicotinamide riboside	0.0307	0.0063	3.99E-06	1.48E-03
1-linoleoylglycerol (18:2)	0.0952	0.0208	1.92E-05	3.93E-03
2,4-di-tert-butylphenol	-0.2822	0.0633	2.24E-05	3.93E-03
N-acetylserine	0.0100	0.0024	6.32E-05	8.57E-03
cytidine 5'-diphosphocholine	-0.0146	0.0035	6.91E-05	8.57E-03
X - 11979	0.0237	0.0060	1.37E-04	1.06E-02
X - 23026	0.1071	0.0270	1.37E-04	1.06E-02
cholate	-0.0445	0.0112	1.43E-04	1.06E-02
heptenedioate (C7:1-DC)*	0.2572	0.0628	1.44E-04	1.06E-02
linolenate [alpha or gamma; (18:3n3 or 6)]	0.0706	0.0181	1.73E-04	1.17E-02
argininosuccinate	0.0302	0.0079	2.43E-04	1.51E-02
linoleate (18:2n6)	0.0857	0.0227	2.83E-04	1.52E-02
5-hydroxylysine	0.0274	0.0072	3.03E-04	1.52E-02
stachydrine	-0.0780	0.0208	3.06E-04	1.52E-02
cystine	0.0490	0.0134	4.25E-04	1.86E-02
docosapentaenoate (n3 DPA; 22:5n3)	0.0524	0.0144	4.26E-04	1.86E-02
trigonelline (N'-methylnicotinate)	-0.2829	0.0803	6.61E-04	2.59E-02
1-ribosyl-imidazoleacetate*	0.0542	0.0154	6.64E-04	2.59E-02
pyridoxate	0.1150	0.0342	1.17E-03	4.29E-02
12-HETE	0.0298	0.0088	1.21E-03	4.29E-02
eicosapentaenoate (EPA; 20:5n3)	0.0403	0.0122	1.31E-03	4.42E-02

Table S11 Summary of data sources used

	Study Description Link	Number of samples	Notes
ROS & MAP cohorts	General documentation: https://www.radc.rush.edu/documentation.htm	2086	Available upon application
	RNA-seq data (DLPFC) https://www.synapse.org/#!Synapse:syn3388564	373	Available upon application
	DNA-methylation data https://www.synapse.org/#!Synapse:syn3157275	431	Available upon application
	Bile Acid concentrations https://www.synapse.org/#!Synapse:syn10235594	74	Available upon application
	Metabolon metabolomics https://www.synapse.org/#!Synapse:syn25878459	338	Available upon application
Surgical menopause miRNA	GEO accession number: GSE194086	22	Publicly available
Age at Menopause-UKBB	https://gwas.mrcieu.ac.uk/datasets/ukb-b-17422/	143,819	Publicly available
Age at Menopause-Ruth et al.	https://www.reprogen.org/data_download.html	201,323	Publicly available
